# Supplementary material for: Searching for simple rules in Pseudomonas aeruginosa biofilm formation
Source: BMC Res Notes. 2019 Nov 21;12:763. doi: 10.1186/s13104-019-4795-x (PMC6873713; doi:10.1186/s13104-019-4795-x)
Supplement: Supplementary file 1 — Additional file 1: Table S1. CA model parameters for fitting WT and WT+AZM. [file 13104_2019_4795_MOESM1_ESM.docx]

Table S1. CA model parameters for fitting WT and WT+AZM

| CA Model | WT | WT+AZM |
| --- | --- | --- |
| Rule i) *X_1_* | 2 | 2 |
| Rule ii) *X_2_* | 2 | 2 |
| Rule ii) *X_3_* | 3 | 3 |
| Rule iii) *X_4_* | 3 | 3 |
| Rule iv) *X_5_* | 3 | 3 |
| Rule iv) *X_6_* | 3 | 3 |
| % motile cells | 100 | 0 |
